# A Rare Case of Post-Traumatic Posterior Chamber Intraocular Lens Extrusion Through the Scleral Tunnel of Manual Small Incision Cataract Surgery

**DOI:** 10.7759/cureus.42884

**Published:** 2023-08-03

**Authors:** Faizan Luqman, Vaneeza Qureshi, Ayma Asad, Muhammad A Khizer, Marwa Mukhtar

**Affiliations:** 1 Ophthalmology, Medical Teaching Institution (MTI) Khyber Teaching Hospital, Peshawar, PAK; 2 Ophthalmology, Khyber Medical College, Peshawar, PAK; 3 Ophthalmology, National University of Medical Sciences, Rawalpindi, PAK; 4 Ophthalmology, Armed Forces Institute of Ophthalmology, Rawalpindi, PAK; 5 Ophthalmology, Medical Teaching Institution (MTI) Ayub Teaching Hospital, Abbottabad, PAK

**Keywords:** subconjunctival pc-iol, manual small‑incision cataract surgery, scleral tunnel, blunt ocular trauma, msics, extruded pc-iol

## Abstract

Traumatic posterior chamber intraocular lens (PC-IOL) extrusion via a self-sealing scleral tunnel, created for manual small-incision cataract surgery (MSICS), is a rare occurrence that has never been reported before. Usually, the PC-IOL protrudes through a ruptured cornea or falls back into the vitreous after blunt trauma. Here, we present a case of PC-IOL extrusion along the uveal tissue through the scleral tunnel in an 80-year-old woman with a history of MSICS who fell and hit her right eye on the stairs, resulting in sudden and painful loss of vision in the same eye. The IOL, along with necrotic uveal tissue, was removed from the subconjunctival space, and the ruptured scleral tunnel was sutured. After initial management, her best-corrected visual acuity (BCVA) was 6/36 with aphakic spectacle correction. The patient was advised to undergo secondary scleral fixation of the intraocular lens. The scleral tunnel made in MSICS is a potentially weak area, and the PC-IOL can come out through it. Therefore, suturing the scleral tunnel with a non-absorbable nylon 10-0 suture should be considered during MSICS. This provides additional support to the weakened scleral wall.

## Introduction

Traditionally, phacoemulsification (phaco) has been the standard procedure for cataract surgery. However, it has some disadvantages, such as a longer duration of surgery, higher level of expertise requirement, unavailability of the phaco machine everywhere, and difficulty in extracting brunescent cataracts. Due to these issues, manual small incision cataract surgery (MSICS), an alternate cataract extraction surgical technique, is gaining popularity in developing countries [1]. It is a sutureless technique and completed through a superior or temporal incision; the backbone of MSICS lies in building a self-sealing sclera-corneal tunnel from which the cataractous lens is delivered [2]. MSICS has advantages, but it does not come without risks. As this procedure involves manipulation of the anterior chamber during nucleus delivery, intraoperative complications include iatrogenic iris trauma, striate keratopathy, and posterior capsular rupture, while postoperative complications include inflammation and corneal edema [3]. Here, we present a rare case of posterior chamber intraocular lens (PC-IOL) extrusion through the scleral tunnel created during MSICS after blunt trauma as an unexpected complication of MSICS. In this case report, we investigate the complication and its management.

## Case presentation

An 80-year-old female presented to the ophthalmology department of Khyber Teaching Hospital, Peshawar, Pakistan with sudden and painful loss of vision in her right eye after falling on the stairs. Her medical and surgical records indicated that she had undergone bilateral MSICS with PC-IOL placement a year ago at another ophthalmology center. The right eye was operated on first with a PC-IOL, followed by the left eye a few months later. During follow-up after right eye surgery, she had early posterior capsular opacification (PCO) in her right eye; however, her best-corrected visual acuity (BCVA) was 6/12. YAG laser capsulotomy was performed, and the BCVA improved to 6/9. Her last follow-up was five months back, which was unremarkable and she was happy with the vision in both eyes.

**Figure 1:**
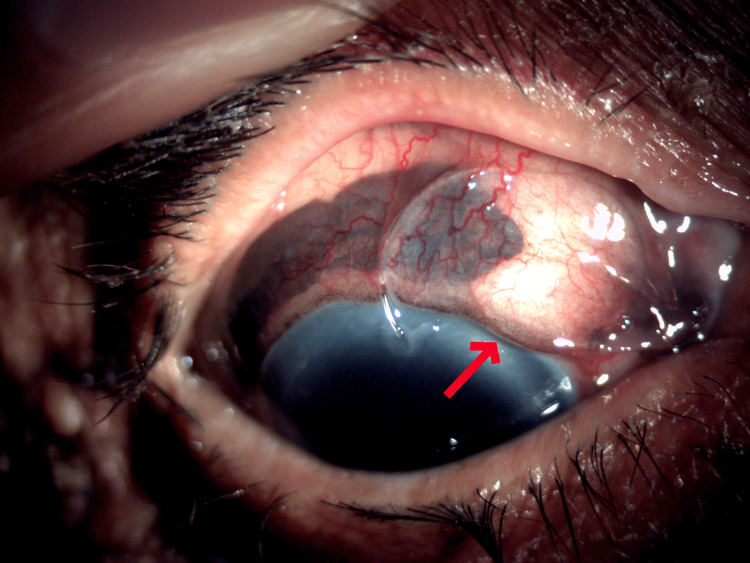
Slit lamp anterior segment photograph showing the extruded PC-IOL (red arrow) along with uveal tissue in the subconjunctival space. PC-IOL: Posterior chamber intraocular lens

**Figure 2:**
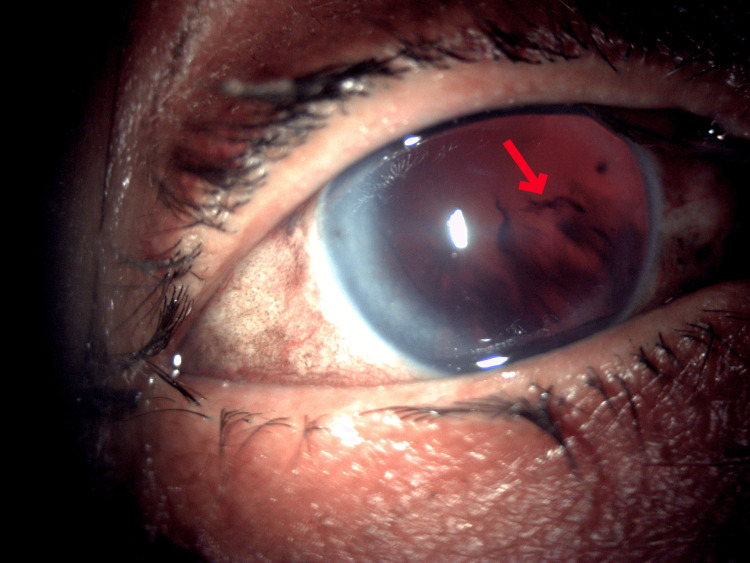
Slit lamp anterior segment photograph taken with retro-illumination showing vitreous haemorrhage (red arrow) and aphakia.

Following the injury to her right eye, the patient noticed a sudden and painful loss of vision. Upon examination, her BCVA was the detection of hand movements. A scleral rupture involving the scleral tunnel with uveal prolapse from the 10 o’clock to the 1 o’clock position was observed with an extruded PC-IOL under the conjunctiva in the superonasal region (Figure 1). The PC-IOL reached the site during trauma via the scleral tunnel. A significant vitreous hemorrhage was also observed in the right eye (Figure 2). Her left eye was pseudophakic, with a BCVA of 6/12. A relative afferent pupillary defect (RAPD) was detected in the left eye, which was attributed to a previously undiagnosed optic neuropathy.

During admission, the patient was treated symptomatically with systemic painkillers, topical antibiotics, steroids, and antiglaucoma medications. The primary repair was performed under general anesthesia. The extruded IOL was removed along with the necrotic uveal tissue from the subconjunctival space. The scleral rupture was repaired using a 10-0 nylon suture. Posterior segment examination revealed significant inferonasal vitreous haemorrhage; however, the rest of the posterior segment was unremarkable. The patient was discharged and referred to a vitreoretinal surgeon for further management. Vitreous haemorrhage was managed via pars plana vitrectomy of the right eye; however, the patient refused scleral fixation of intraocular lens (SF-IOL). Her final BCVA of the right eye was 6/36 with spectacle correction of +10.00 DS/-3.50DC at 115°. She was followed up after a month. Her eye wounds improved, and there was no change in the visual acuity in her right eye. 

## Discussion

Among the available surgical options for cataract extraction, MSICS remains popular in the developing world. Blunt trauma inflicted on the eye damages the IOL itself, causing displacement or expulsion, along with possible corneal damage. Displacement usually occurs in the anterior chamber and posteriorly in the vitreous cavity, although posterior chamber IOL displacement is rare and atypical. Factors such as old age, pre-existing incision scars from previous surgeries, and connective tissue disorders increase the risk of lens displacement following blunt trauma [4]. 

The patient presented here underwent cataract extraction using MSICS a year prior. Following blunt trauma to the eye, the patient presented with a PC-IOL displaced under the conjunctiva through the ruptured scleral tunnel into the superonasal quadrant. We could not ascertain the exact cause of this event because the patient had no local or systemic history that could have potentially weakened the sclera. Possible causes include premature entry by the surgeon during MSICS or other intraoperative complications. The scleral tunnel made during MSICS is a potentially weak area; hence, surgeons should consider using 10-0 nylon sutures to close the scleral tunnel in high-risk patients to prevent post-truamatic extrusion of PC-IOL into the subconjunctival space. Subconjunctival IOL dislocation, which has been previously reported, occurs through sutured limbal wounds [5]. However, in our case, the PC-IOL was displaced subconjunctivally through the scleral tunnel, which has never been reported.

## Conclusions

This complication should be considered during cataract surgery with MSICS. To prevent this complication, surgeons should avoid premature entry into the anterior chamber and consider using sutures with good tensile strength to close the scleral tunnel after MSICS. High-risk patients should be paid extra attention and scheduled for frequent follow-ups.
